# Draft Genome Sequences of Two Bulgarian *Bacillus anthracis* Strains

**DOI:** 10.1128/genomeA.00152-13

**Published:** 2013-04-18

**Authors:** Dawn N. Birdsell, Markus Antwerpen, Paul Keim, Matthias Hanczaruk, Jeffrey T. Foster, Jason W. Sahl, David M. Wagner, Gregor Grass

**Affiliations:** Center for Microbial Genetics and Genomics, Northern Arizona University, Flagstaff, Arizona, USAa;; Pathogen Genomics Division, Translational Genomics Research Institute, Flagstaff, Arizona, USAb;; Bundeswehr Institute of Microbiology, Munich, Germanyc

## Abstract

*Bacillus anthracis* strains previously isolated from Bulgaria form a unique subcluster within the A1.a cluster that is typical for isolates from southeastern Europe. Here, we report the draft genome sequences of two Bulgarian *B. anthracis* strains belonging to the A branch (A.Br.)008/009 canonical single nucleotide polymorphism (SNP) group of the major A branch.

## GENOME ANNOUNCEMENT

Anthrax is a zoonotic disease that is endemic in many countries worldwide. The Gram-positive endospore-forming bacterium *Bacillus anthracis* produces several virulence factors required for establishing infections in animal and human hosts. Major virulence factors are encoded on two large plasmids, pX01 and pX02. Despite the importance of these plasmids for host infections, many strains of *B. anthracis*, especially those isolated from soil and/or after long-term culturing, lack one or both plasmids (1). Previously, we genetically analyzed 40 *B. anthracis* strains collected in Bulgaria, isolated mostly between 1960 and 1980, and found that over half of them lacked one or both plasmids (2). The three major phylogenetic lineages (branches A, B, and C) of *B. anthracis* are divided into 12 clonal groups by the analysis of 13 canonical single nucleotide polymorphisms (canSNPs) (3). We classified each strain as belonging to either the A branch (A.Br.)008/009 or A.Br.WNA genetic group (2) and placed all 40 strains onto the existing global phylogenetic tree (3, 4). As part of our continuing efforts aimed at determining the phylogeography of *B. anthracis* isolates in Europe, we sequenced the genomes of two Bulgarian *B. anthracis* strains representing the A.Br.008/009 genetic group. Whole genome sequences of these strains permit in-depth comparisons of Bulgarian *B. anthracis* A.Br.008/009 strains with other A.Br.008/009 strains from western and central Europe and areas south and east of the Black Sea (2).

Whole-genome shotgun (WGS) sequencing of the *B. anthracis* strains was performed using the Illumina GAIIx sequencing platform (Illumina, Inc.). For the WGS libraries, 4.6 to 5.79 million 100-bp reads were generated. Reads were assembled with AMOScmp (5), using the *B. anthracis* Ames ancestor strain (6) as the reference. Following assembly, contigs were processed with the PAGIT pipeline (7). A *de novo* assembly was also performed with Velvet (8), in conjunction with the VelvetOptimiser (http://bioinformatics.net.au/software.velvetoptimiser.shtml). The AMOS and Velvet assemblies were then aligned with Mugsy (9). Regions unique to the *de novo* assembly were then aligned back against the comparative assembly with BLASTn (10). Contigs that failed to align were concatenated with the comparative assembly into the final assembly. The assembled contigs were submitted to the RAST annotation server for subsystem classification and functional annotation (11).

The total lengths of the draft genome shotgun sequences of *B. anthracis* strains 3154 and 3166 were 5.0 and 5.5 Mbp, respectively, and their mean G+C content was 35.35% ± 0.1%. The chromosomal sequences of these strains contained 3 to 5 contigs with 189- to 235-fold average coverage. The plasmids pX01 and pX02 in strain 3166 were assembled on 2 contigs with 434-fold coverage. The *B. anthracis* 3154 genome contains a ~200-kb deletion compared to the Ames ancestor (accession no. YP_019942 to YP_020195), comprising genes for a beta-lactam antibiotic acylase (accession no. YP_019964) and a sulfamethoxypyridazine (SMPR) multidrug efflux pump (accession no. YP_019986).

### Nucleotide sequence accession numbers. 

The draft genome sequences of *B. anthracis* strains 3154 and 3166 have been included in the GenBank WGS database under accession no. ANFF00000000 and ANFG00000000, respectively. The versions described in this paper are the first versions, accession no. ANFF01000000 and ANFG01000000, respectively.
